# Taking OSCE examiner training on the road: reaching the masses

**DOI:** 10.3402/meo.v21.32389

**Published:** 2016-09-28

**Authors:** Katharine Reid, David Smallwood, Margo Collins, Ruth Sutherland, Agnes Dodds

**Affiliations:** Department of Medical Education, Melbourne Medical School, The University of Melbourne, Victoria, Melbourne, Australia

**Keywords:** objective structured clinical examinations, medical education, examiner training, assessment, reliability

## Abstract

**Background:**

To ensure the rigour of objective structured clinical examinations (OSCEs) in assessing medical students, medical school educators must educate examiners with a view to standardising examiner assessment behaviour. Delivering OSCE examiner training is a necessary yet challenging part of the OSCE process. A novel approach to implementing training for current and potential OSCE examiners was trialled by delivering large-group education sessions at major teaching hospitals.

**Methods:**

The ‘OSCE Roadshow’ comprised a short training session delivered in the context of teaching hospital ‘Grand Rounds’ to current and potential OSCE examiners. The training was developed to educate clinicians about OSCE processes, clarify the examiners’ role and required behaviours, and to review marking guides and mark allocation in an effort to standardise OSCE processes and encourage consistency in examiner marking behaviour. A short exercise allowed participants to practise marking a mock OSCE to investigate examiner marking behaviour after the training.

**Results:**

OSCE Roadshows at four metropolitan and one rural teaching hospital were well received and well attended by 171 clinicians across six sessions. Unexpectedly, medical students also attended in large numbers (*n=*220). After training, participants’ average scores for the mock OSCE clustered closely around the ideal score of 28 (out of 40), and the average scores did not differ according to the levels of clinical experience.

**Conclusion:**

The OSCE Roadshow demonstrated the potential of brief familiarisation training in reaching large numbers of current and potential OSCE examiners in a time and cost-effective manner to promote standardisation of OSCE processes.

Since first described by Harden and Gleeson (1), objective structured clinical examinations (OSCEs) have become widely used in medical education, both in routine assessments of medical student competence and in high stakes examinations (2). Medical schools invest significant resources in designing and implementing OSCE in assessment programmes, with the rigour of the process highly dependent on whether OSCEs provide reliable and valid indicators of student competence (3). OSCEs aim to decrease extraneous variability through standardising aspects of the assessment including instructions to students, examiner questions, standardised simulated patient scripts, uniform clinical materials and through developing detailed assessment check lists. Despite such measures, significant variation between examiners in OSCE marking remains (4).

Due to concerns that examiner variation might affect assessment reliability, educators have proposed various approaches to improving the standardisation of OSCE procedures. These approaches include examiner training, which has been advocated as an integral component of OSCE processes and a means to promote the consistency of OSCEs (5–7). Yet, data on how training affects examiners’ ratings of clinical competence are limited. Research suggests that untrained clinical examiners may be less consistent and more lenient than trained examiners (8, 9); however, Cook et al. (10) found that a half-day examiner training workshop did not significantly improve reliability and accuracy of Mini-CEX ratings. Training of OSCE examiners also presents significant challenges. Examiners’ clinical responsibilities leave limited time for training, and many are unable to attend case-specific training sessions; thus, a uniform approach to examiner training is a constant challenge.

Practical solutions to OSCE examiner training are needed, which address some of these difficulties while balancing resourcing constraints. This article reports on an approach to educating clinicians about the OSCE process as part of an ongoing commitment to improving the reliability of OSCEs. The approach aimed to reach a wide range of clinicians (both new and experienced OSCE examiners) by providing training in a general educational forum. Such training provided an opportunity to orient clinicians to the OSCE process and served as an adjunct to (and not a replacement for) detailed case-specific training conducted prior to OSCEs. Overall, the intervention aimed to reach large numbers of current and potential OSCE examiners in an accessible format, to educate them about OSCE processes in an effort to improve and standardise examiner behaviour in OSCEs.

## Method

The impetus for developing this OSCE training and decisions on the content of the training evolved from discussions in the OSCE Committee about inconsistencies in examiner behaviour. An educational process was sought which clarified the purpose of OSCEs and reinforced expected examiner behaviour. The major teaching hospitals for our medical school have weekly ‘Grand Rounds’. Grand Rounds are clinical meetings where medical students, junior medical officers, and senior medical staff gather to present recent medical research, ‘bench to bedside’ evidence, and interesting clinical cases. These meetings are highly regarded and generally well attended. Using these forums provided opportunities to raise the profile of medical education generally and to provide specific information about the purpose and philosophical underpinnings of OSCEs. The sessions were named the OSCE Roadshow because they involved touring different teaching hospitals to deliver the training.

A single presenter (a physician who was the chair of the OSCE Committee) delivered all the training, which, across approximately 1 h, described how OSCE stations are developed, explained the examiners’ role, and defined appropriate examiner behaviour. The training initially focused on the principles of clinical assessment and the rationale for using OSCEs, including their advantages and disadvantages. The training highlighted methods of OSCE construction, particularly the use of diagnostic algorithms in designing the marking sheets, and focused on how to allocate OSCE checklist marks and global scores. Towards the end of the training, participants undertook an exercise that involved viewing and scoring a 9-min mock OSCE station, designed specifically for this educational exercise, on DVD. The diagnostic interview OSCE showed actors portraying a female medical student conducting an interview with a male patient presenting with dysphagia. This exercise allowed a simple, preliminary check of the concordance between actual and ideal scoring of the mock OSCE and of the consistency across attendees with different clinical experience after undertaking the training.

The diagnostic interview OSCE developed for the training was developmentally appropriate for a student at the end of 1 year of clinical training and matched the current format of a 9-min OSCE with 1 min for examiner questions (a ‘post encounter probe’). Content experts wrote the script for the simulated patient and the marking scheme; these followed evidence-based diagnostic algorithms. The marking schema for diagnostic interview OSCEs at our medical school follows a scheme inductive reasoning approach, with increased weighting given to important features and key decision points. Participants marked the OSCE by assigning marks according to a checklist in the following six sections: clarifies the presenting problem (7 marks), asks about the time course and associated features (7 marks), asks about the risk factors for oesophageal disease (6 marks), asks about symptoms suggestive of malignancy (5 marks), a global communication skills mark (5 marks), and responses to examiner questions (10 marks). OSCE checklists are detailed with individual marks awarded to demonstrated diagnostic interviewing skills within these broader sections.

The OSCE was tightly scripted so that the ‘correct’ score for the OSCE was 28 out of 40 possible marks. The student's performance was designed to be clearly at a satisfactory level after 1 year of clinical training. The actor playing the examiner asked three questions after 8 min. OSCE Roadshow participants scored the student's performance individually, using the structured mark sheet, by scoring skills within the six sub-sections and also providing a global performance rating, using a three-point rating scale where 1=unsatisfactory, 2=borderline, and 3=satisfactory. At the end of the OSCE, marks were tallied and the sheets collected. Participants were asked to indicate by raising their hands what marks they had allocated, in order to provide immediate feedback and promote discussion. A short presentation regarding the characteristics of a ‘good examiner’ and a description of the pathways that examiners can use to provide feedback on OSCEs that they examine concluded the session. Audience members had an opportunity to ask questions in the final 5–10 min of the session.

## Results

Participants attended the OSCE Roadshow at one of five medical and one surgical Grand Rounds conducted across five clinical training hospitals (four in metropolitan locations and one in a rural location). The OSCE Roadshow was well received with 171 clinicians in total attending one of the six training sessions. Participants comprised senior medical staff (*n=*83), registrars (*n=*54), residents (*n=*19), and interns (*n=*15). The majority of attendees were male (70.9%), with the average age of participants approximately 40 years. There was also significant interest from medical students (*n=*220) at every session. Other health professionals (*n=*31) also attended some sessions. Only registered medical practitioners were considered in these results as our focus was on those currently examining OSCEs or those who were eligible to examine during the next assessment period.

To explore marking behaviour after the OSCE training, we assessed the degree to which participants’ scores varied from the ‘ideal’ score of 28 marks out of 40. The majority of total scores (80.7%) ranged from 26 to 30, and 89.5% ranged from 25 to 31. Total scores outside the range 26–30 were no more or less likely according to participants’ professional role or their gender. Almost all participants who provided global ratings indicated that performance on the OSCE was satisfactory (98.1%).

Table 1 shows mean scores (and standard deviations) according to participants’ professional role. These data suggest minimal variation in average scores for participants with different levels of experience. To examine variation in scoring as a function of participant gender and their professional role, we conducted a 2 (gender)×4 (professional role) analysis of variance with total score on the dysphagia OSCE as the dependent measure. Average scores for male and female participants did not vary, *F* (1, 157)=0.12, *p=*0.729, and participants with different professional roles also scored the OSCE similarly, *F* (3, 157)=1.34, *p=*0.263. Table 2 shows high agreement in marking across the six sections of the marking scheme.

**Table 1:** Descriptive statistics for the dysphagia OSCE total score by participant type

	*M*	SD	*N*
Interns	28.2	0.94	15
Residents	28.4	1.74	19
Registrars	28.8	2.20	83
Senior medical staff	29.0	1.97	54
Total	28.7	2.00	171

**Table 2:** Distribution of OSCE section marking around ideal section scores (%)

OSCE section (maximum score)	OSCE section score marks										

	0	1	2	3	4	5	6	7	8	9	10
Clarifies the nature of the presenting problem (7)					1.2	1.8	43.3	53.8			
Asks about time course and associated features (7)			1.2	4.1	7.0	66.1	16.4	5.3			
Asks about history of risk factors for oesophageal disease (6)				1.8	3.5	72.5	22.2				
Asks about symptoms suggestive of malignancy (5)	0.6	86.5	9.4	2.9		0.6					
Communication skills mark (5)			1.8	24.6	53.2	20.5					
Examiner questions (10)				1.2		6.4	5.3	78.4	2.3	6.4	

*Note*. The percentage frequency of scores allocating the ‘ideal’ section score are noted in the table with dark grey shading.

## Discussion

OSCEs are a central component of assessing medical students’ clinical skills, and because such assessments inform decisions on clinical competence, it is vital to ensure that OSCE processes are rigorous. However, prior research suggests that examiner marking behaviour is a known source of variation in OSCE scoring (11–13), despite significant efforts to standardise features of the examination (4). Approaches to dealing with such variation have included identifying and remediating examiners awarding aberrant scores–so-called hawks and doves (14), but by and large examiner training is advocated as a key element of standardising examiner marking behaviour.

Although training is consistently advocated as a central feature of the OSCE process (5–7), descriptions of training approaches and evidence of their effect are lacking. The OSCE Roadshow described in this article trialled a novel training approach to guide potential OSCE examiners to better understand the purpose of OSCEs and to mark OSCEs consistently. The approach employed weekly Grand Rounds as a training forum. Participants received training on OSCE development and how OSCEs are used to assess clinical skills, and they practised marking a specifically developed OSCE scenario presented on DVD. The OSCE Roadshow was well received and well attended with 171 clinicians in total present across the six sessions, suggesting these forums are a means to reach large number of clinicians and to enable clinicians with significant clinical responsibilities to attend.

OSCE examiner training is often recommended in general terms as a means of reducing examiner variability (15), or researchers may describe the characteristics of structured OSCE training programs (7). Yet as outlined, research on the effects of OSCE training on examiner marking behaviour is rare and has shown inconsistent effects. For instance, a longer (2.5 h) and more individualised (four to six participants) OSCE training session evaluated by Byrne et al. (16) appeared to have no effect on measures of examiner error or mental workload. In contrast, Schwartzman et al. (17) demonstrated that short pre-OSCE training sessions removed variation between clinician and simulated patient (SP) examiners in communication skills ratings on a pharmacy OSCE. Our findings suggest a high degree of concordance in examiner marking of the mock OSCE to an accurate standard after a short large-group training session. The training thus showed promise in reducing known variability in examiner marking with participants’ marking of the mock OSCE clustered closely around the ideal total score of 28 out of 40. Moreover, almost all clinicians recognised that the performance met the required standard for a student at the end of 1 year of clinical training.

Interestingly, clinicians with different levels of experience scored the OSCE similarly, which supports findings suggesting that training reduces variation in the marking of more and less experienced examiners (18). Our results are similar to those of Schwartzman et al. (17) on the effects of training in aligning the ratings of skilled clinicians and SP raters, suggesting that brief training may have merit in reducing examiner variation due to marking experience. One relevant consideration, however, is that the mock OSCE developed for this research to illustrate post-training examiner behaviour was at a clearly satisfactory level. Byrne et al. (16) showed increased examiner error in OSCE marking for borderline performances compared with clearly unsatisfactory or outstanding performances. Thus, the satisfactory performance shown in our mock OSCE could be a less cognitively demanding task, which may have supported more and less experienced clinicians to mark the OSCE similarly.

An unanticipated outcome was the large number of medical students who attended Grand Rounds during the OSCE Roadshow. Across the six sessions, 220 medical students attended the presentations. This unexpected interest from students suggests potential for educating the wider student body about the OSCE process. Developing medical students’ skills as future educators has also been advocated (19), and the high level of attendance by medical students at the OSCE Roadshow suggests this context could be an appropriate forum for this to occur.

## Limitations

There is, however, a need to examine whether these results are reflected in OSCEs more generally. The OSCE Roadshow obviously did not replicate the conditions of OSCE examination; participants had only a few minutes to review the OSCE mark sheet prior to watching the OSCE, and although participants were told not to confer with colleagues, it is possible that this occurred. Ideally, our real-world scenario might have included a measure of examiner marking behaviour on an OSCE presented at the beginning of the training session, as a means to more precisely determine the impact of the training. The evidence that it is more difficult for examiners to be consistent for borderline performances (16), suggests that comparing performance on OSCEs of varying standards would be a more stringent test of the usefulness of the training.

## Conclusions

Medical schools are consistently challenged by the need to engage clinicians to participate in University education and examination of medical students. The OSCE Roadshow was shown to be an effective means to engage with current OSCE examiners and hopefully also with a new generation of potential OSCE examiners. The OSCE Roadshow reached a significant number of potential OSCE examiners and focused primarily on promoting clinician engagement with the assessment process, but also targeted the reliability and consistency of OSCE examiner marking. Such training could provide a useful adjunct to case-specific training run prior to OSCEs and, more recently, online compulsory OSCE training resources developed for OSCE examiners. Although previous research has suggested an inconsistent effect of brief examiner training (10, 16), the findings of this study are encouraging, suggesting that a brief, standardised training may help to reduce marking variability in OSCEs. Nonetheless, it is necessary to determine whether these presentations have made a difference to examiner behaviour during OSCEs or have impacted on the reliability and consistency of examiner marking.
